# Induction of Intracellular Ca^2+^ and pH Changes in Sf9 Insect Cells by Rhodojaponin-III, A Natural Botanic Insecticide Isolated from *Rhododendron molle*

**DOI:** 10.3390/molecules16043179

**Published:** 2011-04-15

**Authors:** Xing-An Cheng, Jian-Jun Xie, Mei-Ying Hu, Yan-Bo Zhang, Jing-Fei Huang

**Affiliations:** Laboratory of Insect Toxicology, South China Agricultural University, Guangzhou 510642, China; Email: anzai_28@163.com (X.-A.C); 245738742@qq.com (J.-J.X); fiend_wing_bo@yahoo.com.cn (Y.-B.Z); huangjingfei@163.com (J.-F.H)

**Keywords:** rhodojaponin-III, intracellular free calcium, intracellular pH, Sf9 cells arrest

## Abstract

Many studies on intracellular calcium ([Ca^2+^]_i_) and intracellular pH (pH_i_) have been carried out due to their importance in regulation of different cellular functions. However, most of the previous studies are focused on human or mammalian cells. The purpose of the present study was to characterize the effect of Rhodojaponin-III (R-III) on [Ca^2+^]_i_ and pH_i_ and the proliferation of Sf9 cells. R-III strongly inhibited Sf9 cells proliferation with a time- and dose-dependent manner. Flow cytometry established that R-III interfered with Sf9 cells division and arrested them in G2/M. By using confocal scanning technique, effects of R-III on intracellular free calcium ([Ca^2+^]_i_) and intracellular pH (pH_i_) in Sf9 cells were determined. R-III induced a significant dose-dependent (1, 10, 100, 200 μg/mL) increase in [Ca^2+^]_i_ and pH_i_ of Sf9 cells in presence of Ca^2+^-containing solution (Hanks) and an irreversible decrease in the absence of extra cellular Ca^2+^. We also found that both extra cellular Ca^2+^ and intracellular Ca^2+^ stores contributed to the increase of [Ca^2+^]_i_, because completely treating Sf9 cells with CdCl_2_ (5 mM), a Ca^2+^ channels blocker, R-III (100 μg/mL) induced a transient elevation of [Ca^2+^]_i_ in case of cells either in presence of Ca^2+^ containing or Ca^2+^ free solution. In these conditions, pH_i_ showed similar changes with that of [Ca^2+^]_i_ on the whole. Accordingly, we supposed that there was a certain linkage for change of [Ca^2+^]_i_, cell cycle arrest, proliferation inhibition in Sf9 cells induced by R-III.

## 1. Introduction

**Figure 1:**
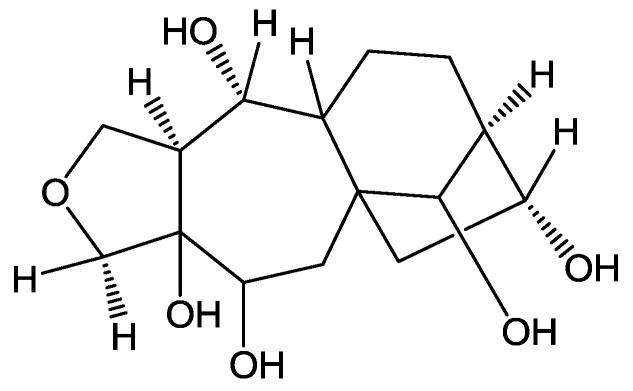
Structure of Rhodojaponin-III.

Rhodojaponin-III (R-III, Figure 1) show structure as Figure 1 is a grayanoid diterpene isolated from *Rhododendron molle* and determined as the main insecticidal ingredient in the plant [1]. It is an effective natural insecticide against more than 40 species of agricultural pests [2]. Previous studies indicate that R-III shows many anti-insect properties including potent antifeedant, oviposition, ovicides, antimolting, growth inhibitor, contact and/or stomach toxicity [3], which is related to the nervous, digestive, endocrine and reproductive systems of insects. There have been some studies on the mode of action of R-III on insects, although the precise molecular mechanism is not well understood. Some researchers demonstrated that R-III remarkably decreases the contents of acetylcholine (ACh) and has reversible activated effects on Na^+^-K^+^-ATPase and Ca^2+^-Mg^2+^-ATPase activities [4], indicating its interference with insect nervous system through blocked the transition of nervous impulse [5], in which Ca^2+ ^as an intracellular second messenger plays a key role.

Intracellular free calcium ([Ca^2+^]_i_) is a highly versatile intracellular second messenger and signal transducer in both excitable and non-excitable cells. It is involved in many functions in proliferative cells, including gene expression, protein synthesis, cell secretion, motility, metabolism, cell-cycle progression and apoptosis cell death [6,7]. Under normal conditions, [Ca^2+^]_i_ concentration is maintained at 10–100 nM, but sustained Ca^2+^ release from intracellular Ca^2+^ stores, Ca^2+^ influx through receptor- or voltage-dependent Ca^2+^ channels or blockage of re-uptake can perturb [Ca^2+^]_i _homeostasis [7]. A variety of physical, chemical, or biological stimuli can modify [Ca^2+^]_i_ which may lead to cellular physiological changes such as cell arrest or cell death [8,9].

Intracellular pH (pH_i_) is becoming evident to many aspects of cell physiology, and protons may also function as a second messenger in a manner similar to that of Ca^2+^ [10]. Relatively small changes in pH_i_ could have a profound effect on a variety of cellular functions. For example, changes in pH_i_ take place in response to growth, tumor promotion, DNA synthesis [11], protein synthesis, activation of the ion channel [12], apoptosis, proliferation and transformation [13]. High pH_i_ can sensibilize cellular proteins such as enzymes, ion channels and ion transporters [14] and pH_i_ shifts may have significant effects on calcium regulation in cells. It has been established in previous research that pH_i_ and [Ca^2+^]_i_ are closely linked. In effect, pH_i_ has been described as being able to affect intracellular Ca^2+^ homeostasis and contribute to the length, magnitude, and frequency of the Ca^2+^ signal through the modulation of voltage-dependent or -independent plasma membrane Ca^2+^ channels and/or through regulation of the mobilization of Ca^2+^ from internal stores [10]. On the other hand, Ca^2+^ has been described as inducing pH_i_ variation, particularly in neurons [15]. In several cellular models cytosolic alkalinization is a sufficient signal to release calcium from intracellular pools [16].

Although many studies on [Ca^2+^]_i_ and pH_i_ have been carried out due to their importance in regulation of different cellular functions, most of the previous studies are focused on human or mammalian cells and similar studies in insect cells are lacking. Studying the mode of action of botanical pesticide against insects has been greatly simplified by the finding that its effects can be seen in cultured insect cells [17,18]. Therefore, the purpose of this study is to principally characterize the effect of R-III on intracellular Ca^2+^ and pH_i_ in Sf9 cells (isolated from *Spodoptera frugiperda *pupal ovarian tissue). Otherwise, we primarily discuss the possible interactions among changes of [Ca^2+^]_i_ level, cell cycle and cell proliferation, and the possible linkage between changes of intracellular Ca^2+^ and that of pH_i_ in Sf9 cells induced by R-III, all of which are helpful to explore some new clues for the further study on insecticidal mechanism of R-III.

## 2. Results and Discussion

### 2.1. Effect of R-III on the Proliferation of Sf9 Cells

**Figure 2:**
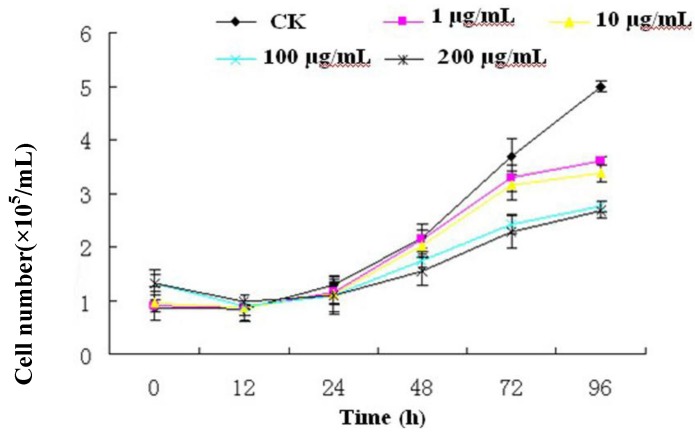
Effects of R-III on the proliferation of Sf9 cells. The cells were grown in presence of 1, 10, 100 and 200 μg/mL of R-III for the times shown in the figure. Survival cell number was counted by means of Trypan blue exclusion with a standard haemocytometer. Each result derived from the mean of three repetitions.

To investigate the effect of R-III on the proliferation of Sf9 cells, cell viability was measured by Trypan blue exclusion assay. As shown in Figure 2, the inhibition effect of R-III on Sf9 cells was not significant and survival cell after 24 h of treatment with 1, 10, 100 and 200 μg/mL of R-III was about 1.0 × 10^5^ cells/mL, similar to that of control. After 24 h of treatments with different concentrations of R-III, cell viability decreased in a time- and dose-dependent manner.

Survival cell number was 3.2 × 10^5^, 3.0 × 10^5^, 2.2×10^5^, 2.1 × 10^5^ cells/mL, which was far lower than that of the control (3.8 × 10^5^ cells/mL) after 72 h of treatment with R-III at the concentrations of 1, 10, 100 and 200 μg/mL respectively. By 96 h of treatments with R-III, survival cell number in control (5.0 × 10^5^ cells/mL) had greatly outstripped the R-III treatment. In addition, the inhibition effect on Sf9 cells of R-III at the concentrations of 100 μg/mL and 200 μg/mL was significantly higher than that of 1 μg/mL and 10 μg/mL. However, the inhibition effect between 100 μg/mL and 200 μg/mL or between 1 μg/mL and 10 μg/mL showed no significant difference. The results in this assay indicated that R-III strongly inhibited the proliferation of Sf9 cells in a time- and dose-dependent manner.

### 2.2. Effect of R-III on Cell Cycle

**Figure 3:**
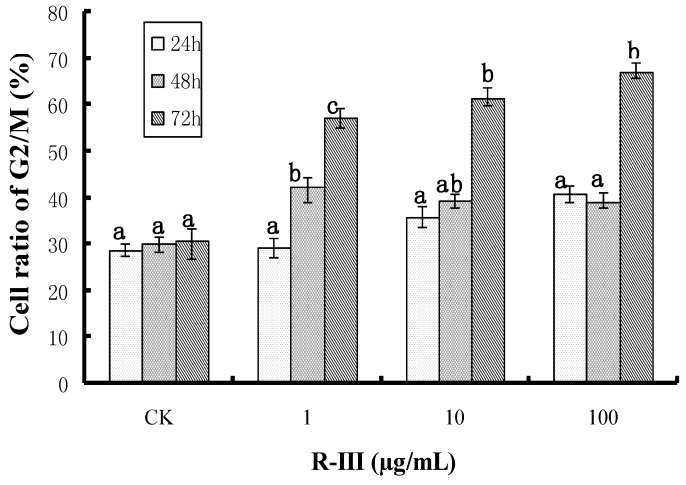
Effects of R-III on cell cycle.The cells were grown in presence of 1, 10, and 100 μg/mL of R-III for the times shown in the figure. Cell cycle was arrested in G2/M in Sf9 cells and showed a time- and dose-dependent manner. Cells that treated with 0.1% DMSO were used as control. The error bars represent mean ± SEM for data derived from three repetitions. Treatment means sharing the same letter were not significantly different from each other (*P < *0*.*05).

In order to further clarify the effects of R-III on the proliferation of Sf9 cells, we checked the effect of R-III on cell cycle by flow cytometry. As shown in Figure 3, an arrest in G2/M cell cycle phase became evident in Sf9 cells treated with R-III, showing a time- and dose-dependent manner. After 24 h of treatment with 1, 10 and 100 μg/mL of R-III, the percentages of cells in G2/M phase increased to 29.2%，35.8%，and 40.7% respectively. While after 48 h of the same treatment, the percent of cells in G2/M phase increased to 42.2%, 39.4% and 39.1% and became 57.1%, 61.3% and 67% respectively, after 72 h of treatment. Comparing to the treated groups, the percent of G2/M phase cells in control was lower (28.6%, 29.9% and 30.5% for 24, 48 and 72 h, respectively) and kept in a steady state within the treated time. Results suggested that R-III like the antimitotic agents such as colchicine and azadirachtin [19] interfered with Sf9 cells division and arrests them in G2/M, showing strong inhibitory activity to the cell growth and proliferation.

### 2.3. Effects of R-III on [Ca^2+^]_i_ and pH_i_ in Sf9 Cells

**Figure 4:**
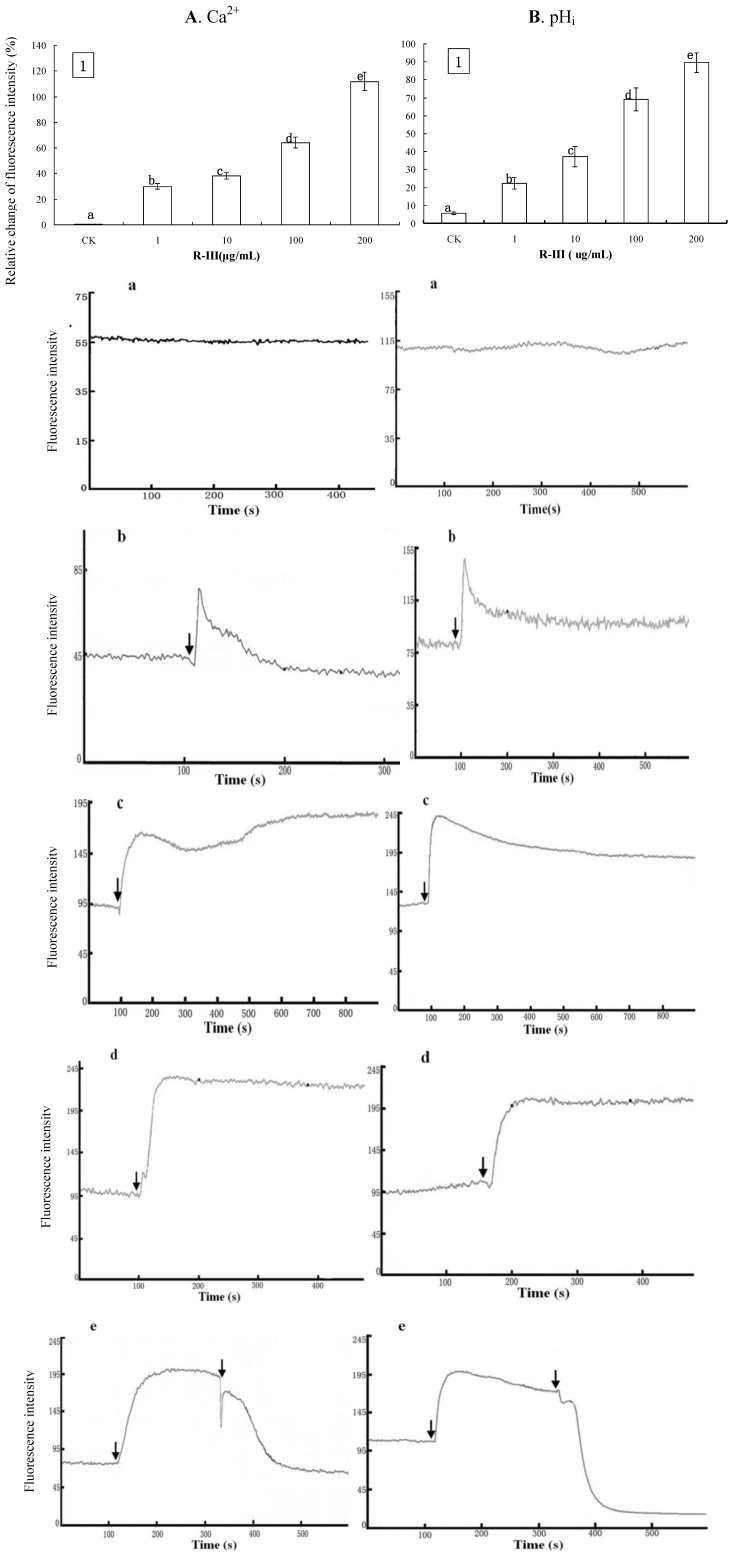
Effect of R-Ⅲ on [Ca^2+^]_i_ and pH_i_ in Sf9 cells in presence of Hanks. (**A1**). changes of [Ca^2+^]_i_ in Sf9 cells stimulated by R-III at various concentrations as indicated by relative change of Fluo-3AM fluorescence intensity; (**B1**). Changes of pH_i_ in Sf9 cells stimulated by R-III at various concentrations as indicated by relative change of Snarf1M fluorescence intensity; (**A a–d**). Dynamic changes of [Ca^2+^]_i_ indicated by a dynamic trace of Fluo-3AM fluorescence intensity in case of Sf9 cells treated with 0, 1, 100, 200 µg/mL of R-Ⅲ respectively; (**B a–d**). pH_i_ profile in cells subject to the protocol in (**A a–d**); (**A-e**). Dynamic variation of [Ca^2+^]_i_ in Sf9 cells treated with 100 µg/mL of R-III for two times; (**B-e**). pH_i_ profile in cells subject to the protocol in (**A-e**). Arrows indicated the addition of R-Ⅲ.Results of representative experiment derived from three repetitions and 4–6 cells were measured in each repetition. The error bars represent mean ± SEM for data derived from value of relative fluorescence intensity in each time interval. Treatment means sharing the same letter were not significantly different from each other (*P < *0*.*05).

In order to know the effects of R-III on [Ca^2+^]_i_, Sf9 cells were exposed to Hanks, a buffer solution containing Ca^2+^. Ca^2+^ influx took place in Sf9 cells stimulated by R-III, which elicited a significant increase of [Ca^2+^]_i_. As shown in Figure 4A1, comparing to control, Sf9 cells showed gradual increase of [Ca^2+^]_i_ by 29.9%, 38.28%, 64.21% and 111.78%, after the stimulation with 1, 10, 100 and 200 μg/mL of R-III respectively, showing a dose-dependent fashion. Under these conditions, we observed a similar change of pH_i_ with that of [Ca^2+^]_i_. As shown in Figure 4B1, Sf9 cells presented a gradual increase of pH_i_ by 22.27%, 37.13%, 69.17% and 89.58% after stimulation with 1, 10, 100 and 200 μg/mL of R-III respectively, also in a dose-dependent fashion. The increase of pH_i_ was only 5.7% in control. In order to further investigate the effects of R-III on [Ca^2+^]_i_ in Sf9 cells, we checked the time-dependent changes of Ca^2+^ fluorescence signals in Sf9 cells induced by R-III. As shown in Figure 4A-a, a flat trace in control indicated no change of [Ca^2+^]_i_ in cells. A transient elevation of [Ca^2+^]_i_ characterized by a fluorescence intensity increase followed by a recovery to basal level was observed in Sf9 cells stimulated with low concentration of R-III (1 μg/mL) (Figure 4A-b), suggesting that cells can regulate [Ca^2+^ ]_i_ to keep intracellular Ca^2+^ homeostasis in case of slight external stimulation.

When exposed to 100 and 200 μg/mL of R-III, Sf9 cells showed a rapid rise of [Ca^2+^]_i_, which reached to a high steady state [Figure 4A-(c,d)]. Previous studies have established that elevation of [Ca^2+^]_i_ may derive from extra cellular Ca^2+^influx through calcium channels or transporters [20] or the Ca^2+^release from intracellular Ca^2+ ^stores induced by intracellular inositol 1,4,5-trisphosphate (IP3), synthesized in response to external stimulation [21]. Under this condition, we applied the second stimulation of R-III, which caused [Ca^2+^]_i_ declining sharply in a index fashion to a steady state lower than the basal level (Figure 4A-e). In this assay, we also found that the changes of pH_i_ were in line with that of [Ca^2+^]_i_, as shown in the traces in Figure 4B-(a,d) and the second stimulation of R-III also produced a sharply decrease of pH_i_ to a steady state lower than the basal level (Figure 4B-e). Although the mechanisms of the sharp decrease of [Ca^2+^]_i_ and pH_i_ are not clear yet, an interpretation from groups of Li *et al.* [22] who investigated the modulation effect of glutamate on [Ca^2+^]_i_ of inner hair cell of the guinea pig cochlea and found similar phenomenon may enlighten us on this study: Excessive stimulation of glutamate may cause toxicity on cells and increase the penetration of plasma membrane (PM) which gives rise to [Ca^2+^]_i_ efflux. Since R-III was a botanic pesticide showing significant toxicity to many kinds of insect, the second stimulation of R-III possibly produced toxic effect on Sf9 cells causing [Ca^2+^]_i_ efflux, in agreement with viewpoint of [22], and [Ca^2+^]_i_ efflux exchanged for H^+^ influx through Ca^2+^-ATPase in PM [23], eliciting decrease of pH_i_.

### 2.4. Effects of R-III on [Ca^2+^]_i_ and pH_i_ in Sf9 Cells in Presence of Dhanks

**Figure 5:**
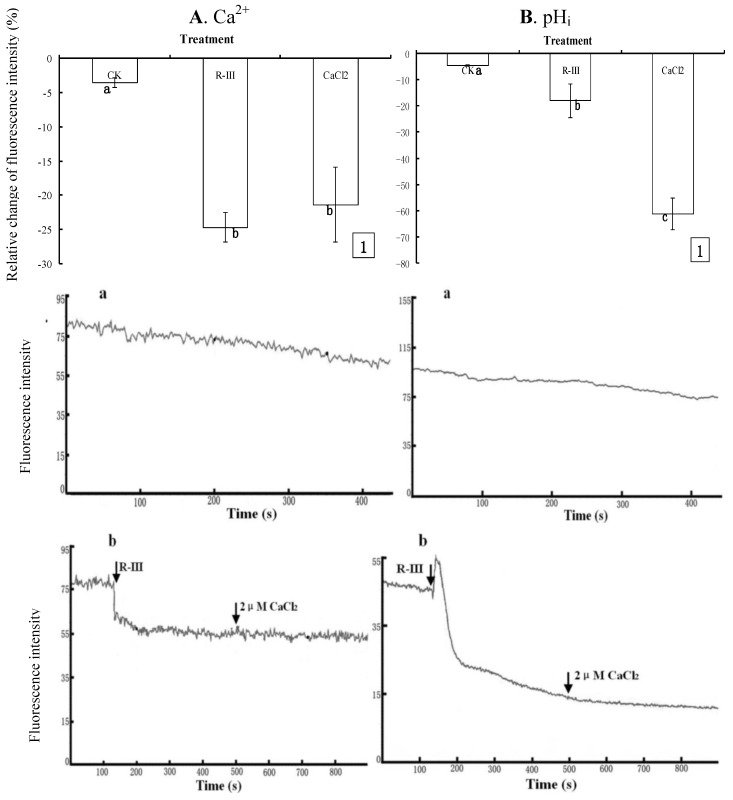
Effects of R-III on [Ca^2+^]_i_ and pH_i_ in Sf9 cells in presence of Dhanks. (**A1**). Changes of [Ca^2+^]_i_ when cells were stimulated by 100 μg/mL in 130 s and subsequent addition of 2 μM CaCl_2_ in 500 s, as indicated by relative change of Fluo-3AM fluorescence intensity; (**A-a**). Control; (**A-b**). Dynamic changes of [Ca^2+^]_i_ in the same conditions with (**A1**); (**B**). pH_i_ profile in cells subjected to the protocol in (**A**). Results of representative experiment derived from three repetitions and 4–6 cells were measured in each repetition. The error bars represent mean ± SEM for data derived from value of relative fluorescence intensity (*vs.* control) in each time interval. Treatment means sharing the same letter were not significantly different from each other (*P < *0*.*05). The negative value meant decrease of relative fluorescence intensity in cells.

To further clarify the effect of R-III on [Ca^2+^]_i_ and pH_i_, Sf9 cells were exposed to Dhanks, a Ca^2+^-free buffer solution, and recoded the change of fluorescence intensity in Sf9 cells stimulated with R-III (100 μg/mL) at 130 s. Comparing to the control with only a slightly decrease of [Ca^2+^]_i_ (3.52%) ( 5A1, A-a), [Ca^2+^]_i_ sharply decreased by 24.64% after the stimulation of R-III (Figure 5A1), and kept in a steady state (Figure 5A-b), indicating [Ca^2+^]_i_ efflux in Sf9 cells induced by R-III, and no increase of [Ca^2+^]_i_ was observed in case of re-addition of CaCl_2_ (2 μM) to the Ca^2+^-free buffer solution at 500 s (Figure 5A-b), suggesting that Ca^2+^ efflux in cells was irreversible. Previous study finds that although glucose oxidase induce a rapid decrease in rat endothelial cells exposed in Ca^2+^ free buffer, re-addition of Ca^2+^ to the extracellular buffer may activate store operated Ca^2+^ entry to cause large [Ca^2+^]_i_ increases [24]. However, store operated Ca^2+^ entry in Sf9 cells was not activated by the re-addition of Ca^2+^ in this assay. The results further proved that it was the Ca^2+^ influx that elicited the substantial increase of [Ca^2+^]_i_ in Sf9 cells stimulated by R-III in case of cell exposure to Hanks in the experiment above [Figure 4A-(b,e)]. Under these conditions, pH_i_ also showed significant decrease (17.85%), and sustained decrease (61.02%) was observed even if addition of CaCl_2_ at 130 s after stimulation (Figure 5B1). Knowing from dynamic change of fluorescence signals in Sf9 cells induced by R-III (Figure 5B-b), R-III induced a transient rise of pH_i_, followed by a decline to a steady level much lower than basal level in Sf9 cells, and no recovery of pH_i_ was observed even if addition of CaCl_2_ at 130 s after stimulation. In contrast, the control showed only a slightly decrease of pH_i_ (Figure 5B-a). Interestingly, [Ca^2+^]_i_ showed no transient increase in the same conditions ( 5A- b). The results in this assay indicated that R-III not only induced [Ca^2+^]_i_ in Sf9 cells decline through Ca^2+^ efflux but also elicited the intracellular acidification, possibly through H^+^ entry in exchange for Ca^2+^ extrusion by the Ca^2+^-ATPase in cell PM [23].

Ca^2+^ signaling plays a crucial role in the function of almost all cell types as an intracellular second messenger [25]. For example, many researches prove that changes in [Ca^2+^]_i_ homeostasis are associated with induction of apoptotic [26] or cell death [27]. An experimental report coming from group of Wang *et al.* [28] provides a convincing interpretation for the role of Ca^2+^ in participation in apoptotic cell death. In their study, the authors found that H_2_O_2_-induced apoptosis of tobacco protoplasts primarily involves in the increase of [Ca^2+^]_i_ resulting from the entry of extra cellular Ca^2+^. In recent years, some reports show that calcium signal is a key component of the molecular switch mechanism in cell division cycle [29].Through the interplay with several proteins, [Ca^2+^]_i_ participates in regulating key steps in the cell cycle such as reentry of quiescent cells into proliferation and the transition through the G1/S, G2/M, and the metaphase/anaphase boundaries [30,31,32]. Moreover, mitosis can be initiated by IP_3_R-induced calcium transients [33]. Disturbance of [Ca^2+^]i homeostasis such as increase of [Ca^2+^]i level in response to external stimulation may interfere with cells division cycle, resulting in cell cycle arrest [34]. In present study, Sf9 cells showed significant changes of [Ca^2+^]i induced by R-III [Figure 4A-(b,e) and Figure 5A-b]. Otherwise, R-III also produced cell cycle arrest in G2/M (Figure 3) and strongly inhibited Sf9 cells proliferation (Figure 2), although apoptosis was not observed. Our results suggested that there was a certain linkage for change of [Ca^2+^]i, cell cycle arrest, cell proliferation inhibition in Sf9 cells induced by R-III. Moreover, we tentatively hypothesize that disturbance of [Ca^2+^]i homeostasis in Sf9 cells induced by R-III may result in cell cycle arrest, which finally causes inhibition of insect cells proliferation or even cell death (including apoptopic cell death). This dual negative effect would significantly decrease the absolute number of cells, and finally induce remarkable decrease of survival cell number in R-III treatment.

### 2.5. The Contribution of Intracellular Ca^2+^ Stores to the Changes of Intracellular Ca^2+^ and pH_i_

**Figure 6:**
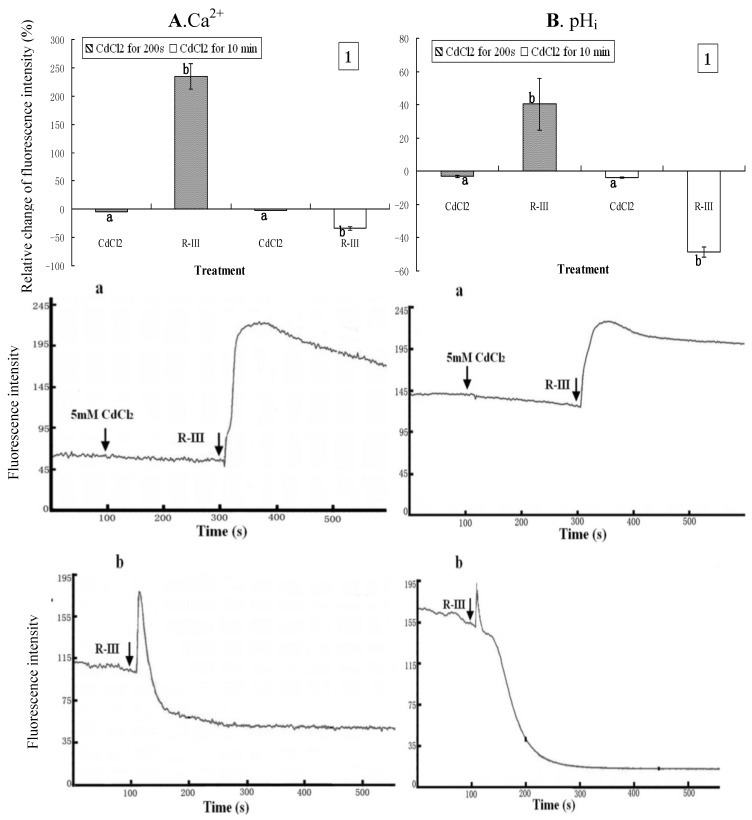
Effect of Ca^2+^ channels blockon [Ca^2+^]_i_ and pH_i_ in R-III-induced Sf9 cells in presence of Hanks. (**A1**). changes of [Ca^2+^]_i_ in Sf9 cells treated with 0.5 mM CdCl_2_ for 200 s and10 min prior to stimulate with R-III (100 μg/mL), as indicated by relative change of Fluo-3AM fluorescence intensity; (**A-a**). Dynamic changes of [Ca^2+^]_i_ in Sf9 cells treated with 0.5 mM CdCl_2_ for 200 s prior to stimulate with R-III (100 μg/mL); (**A-b**). Dynamic changes of [Ca^2+^]_i_ in Sf9 cells incubated with CdCl_2_ (5 mM) for 10 min prior to stimulated with R-III (100 μg/mL); (**B**). pH_i_ profile in cells subjected to the protocol in (**A**). Results of representative experiment derived from three repetitions and 4–6 cells were measured in each repetition. The error bars represent mean ± SEM for data derived from value of relative fluorescence intensity in each time interval. Treatment means sharing the same letter were not significantly different from each other (*P < *0*.*05). The negative value meant decrease of relative fluorescence intensity in cells.

Intracellular Ca^2+^ stores such as mitochondria or endoplasmic reticulum may be the other principal source of Ca^2+^ [35]. In present study, to examine the contribution of intracellular Ca^2+^ stores to the changes of intracellular Ca^2+^, CdCl_2_, a blocker of Ca^2+^ channels was used to treat the cells that were then exposed to Hanks in the following experiments. CdCl_2_ (5 mM) was applied to treat the cells for 200 s prior to the stimulation of R-III (100 μg/mL). As shown in Figure 6A1 and Figure 6B1, [Ca^2+^]_i_ and pH_i_ in Sf9 cells incubated with CdCl_2 _ showed a slight decrease (4.52% and 3.03% respectively). After treatment with R-III, [Ca^2+^]_i_ and pH_i_ rose by 235% and 40.32% respectively. As indicated in dynamic change of trace (Figure 6A-a and B-a), [Ca^2+^]_i_ increased immediately, but followed by a gradual decrease when cells were stimulated by R-III, suggesting that Cd^2+^ gradually blocked the Ca^2+^ channels to inhibit the Ca^2+^ influx. 

Under these conditions, pH_i_ changed in similar fashion with [Ca^2+^]_i_. When we used CdCl_2_ (5 mM) to incubate with Sf9 cells for 10 min to block the Ca^2+^ channels completely, and then stimulated with R-III (100 g/mL), both [Ca^2+^]_i_ and pH_i_ decreased sharply by rate of 33.85% and 48.74% respectively (Figure 6A1 and 6B1). However, in this condition, we found in dynamic change of trace of Figure 6A-b and Figure 6B-b that both [Ca^2+^]_i_ and pH_i_ showed a transient elevation before decreasing sharply. Since Cd^2+^ had blocked Ca^2+^ channels completely and inhibited Ca^2+^ influx, the transient increase of [Ca^2+^]_i_ mainly derived from Ca^2+^ released from intracellular Ca^2+^ stores. It is well established that inositol 1,4,5-trisphosphate (IP3), synthesized in response to external stimulation, induces the release of Ca^2+^ from intracellular stores [21]. In this assay, stimulation of R-III may also induce the synthesis and increase of IP_3_ to promote release of Ca^2+^ from intracellular stores through the Ca^2+^-ATPase. Otherwise, the Ca^2+^ sustained release from intracellular Ca^2+^ stores may likely give rise to its depletion, which could activate store-operated Ca^2+^ channels to promote the Ca^2+^ influx in mammalian non-excitable cells [36], whereas Ca^2+^ channels had been blocked completely by CdCl_2_, and no Ca^2+^ entry but efflux characterized by sharp decline of [Ca^2+^]_i_ to a level far lower than basal level occurred in this study (Figure 6A-b). Under this condition, we observed a proportional change of pH_i_ with that of [Ca^2+^]_i_. We hypothesized that Ca^2+^ released from intracellular stores through the Ca^2+^-ATPase in exchange for H^+^ entry intracellular stores resulted in the transient increase of pH_i_, and the Ca^2+^-ATPase of PM was activated by the transient increase of [Ca^2+^]_i_ and the sustained stimulation of R-III. [Ca^2+^]_i_ effused through Ca^2+^-ATPase in exchange for H^+^ entry intracellular cytosol, which caused the decrease of pH_i_. The results in this assay demonstrated that both extra calcium influx and Ca^2+^ release from intracellular Ca^2+^ stores contributed to the elevation of [Ca^2+^]_i_ in Sf9 cells stimulated by R-III, and pH_i_ showed proportional change with [Ca^2+^]_i_^+^ in response to the stimulation of R-III.

**Figure 7:**
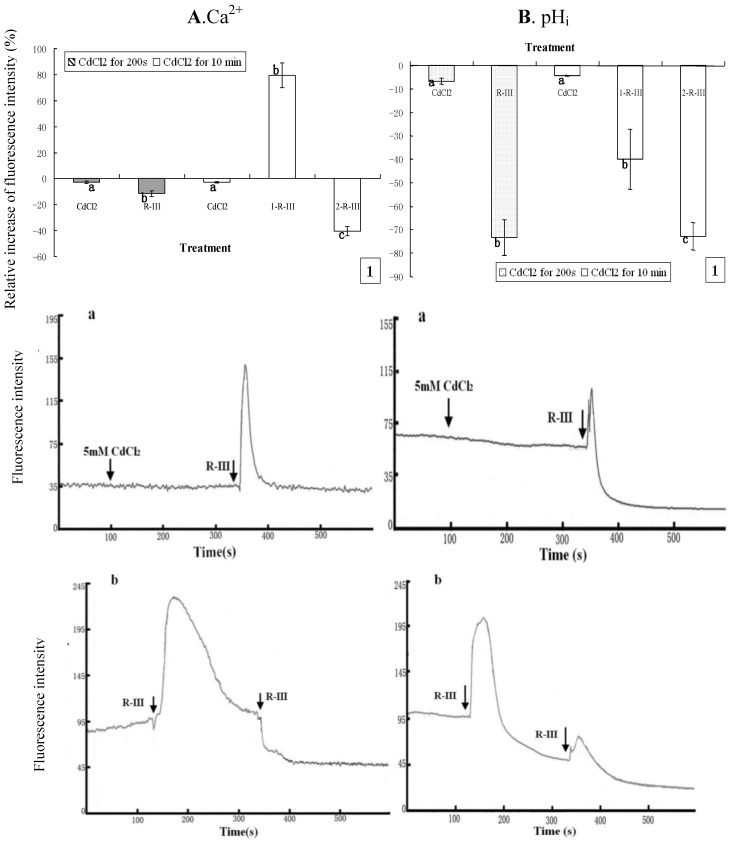
Effect of Ca^2+^ channels block on [Ca^2+^]_i_ and pH_i_ in R-III-induced Sf9 cells in presence of Dhanks. (**A1**). changes of [Ca^2+^]_i_ in Sf9 cells treated with 0.5 mM CdCl_2_ for 200 s and 10 min prior to stimulate with R-III (100 μg/mL), as indicated by relative change of Fluo-3AM fluorescence intensity; (Aa). Dynamic changes of [Ca^2+^]_i_ in Sf9 cells incubated with CdCl_2_ (5 mM) for 200 s prior to stimulate with R-III (100 μg/mL); (**A-b**). Dynamic changes of [Ca^2+^]_i_ in Sf9 cells incubated with CdCl_2_ (5 mM) for 10 min prior to stimulate with R-III (100 μg/mL) for two times; (**B**). pH_i_ profile in cells subjected to the protocol in (**A**). Results of representative experiment derived from three repetitions and 4–6 cells were measured in each repetition. The error bars represent mean ± SEM for data derived from value of relative fluorescence intensity in each time interval. Treatment means sharing the same letter were not significantly different from each other (*P < *0*.*05). The negative value meant decrease of relative fluorescence intensity in cells.

To further clarify the sources of Ca^2+^ and the response of intracellular Ca^2+^ stores in Sf9 cells stimulated by R-III, we repeated the above experiment with the only different condition of cells being exposed to Dhanks. As shown in Figure 7Aand Figure 7B, [Ca^2+^]_i_ and pH_i_ of Sf9 cells indicated only a slight decline in case of incubation with CdCl_2_ for 200s or 10 min. However, the subsequent addition of stimulation by R-III to Sf9 cells after incubation with CdCl_2_ for 200 s gave rise to a dramatic decrease of pH_i_ by rate of 73.36% and only a slight decrease of [Ca^2+^]_i_ (11.55%) ( 7A1 and Figure 7B1). Under this condition, we got the information from Figure 7A-a that [Ca^2+^]_i_ showed only a transient increase followed by a rapid recovery to the basal level, which explained well the only slight change of [Ca^2+^]_i_ in this time interval. Since cells were in presence of Ca^2+^ free solution, and Ca^2+^ channels in PM were at least partially blocked, the transient increase of [Ca^2+^]_i_ should mainly derive from intracellular Ca^2+^ stores, which further prove the contribution of intracellular Ca^2+ ^stores to the changes of intracellular Ca^2+^.

Meanwhile, pH_i_ under these conditions also showed a transient increase similar with that of [Ca^2+^]_i_, but decline finally to a state much lower than the basal level (Figure 7B-a), which induced a high rate of pH_i_decrease (Figure 7A1) differing from the change of [Ca^2+^]_i_. Nevertheless, cells pretreated for 10 min with CdCl_2_ (5 mM) presented an markedly increase of [Ca^2+^]_i_ by rate of 79.43% in the first stimulation of R-III for 200 s, and the second stimulation of R-III induced [Ca^2+^]_i_ decrease by 40.35% (Figure 7A1). Knowing from the dynamic trace in Figure 7A-b, [Ca^2+^]_i_ in Sf9 cells showed a transient elevation followed by a gradual decline to basal level in the first stimulation of R-III, which made the peak of dynamic trace much wider than that of Figure 6A-b, suggesting that [Ca^2+^]_i_ was much higher in this time interval. In contrast, R-III induced a decrease of pH_i_ by rate of 39.85% in first stimulation and 72.78% in the second stimulation (Figure 7B1). We found from Figure 7B-b that pH_i_ in Sf9 cell also showed a transient elevation, but followed by a rapid decline in the first stimulation of R-III. The peak of dynamic trace also became wider comparing to that of Figure 6A-b. Although the reason for these phenomenon was not clear, the results in this assay further indicated that pH_i_ showed proportional change with [Ca^2+^]_i_^+^ in response to the stimulation of R-III on the whole.

It is known that the functional relationships and crosstalk between calcium and pH receive more and more attention, specially, on human cells, but little on insect cells. Although many studies show that changes of pH_i_are associated with that of [Ca^2+^]_i_ in a number of cell types, a clear relationships between the steady state level of pH_i_ and [Ca^2+^]_i_ is not observed in present because interrelationships between pH_i_ and [Ca^2+^]_i_ are rather complex and depend on the cell type [37]. A few studies show that cytosolic alkalinization shift is associated with the increase of [Ca^2+^]_i_ [38] and that acidification shift is associated with the decrease of [Ca^2+^]_i_ [39]. More specifically, an experimental report on crayfish muscle fibre from Kaila and Voipio [40] shows that resting cytosolic calcium is decreased by intracellular alkalosis. In present study, the changes of pH_i_ in Sf9 cells induced by R-III show distinct proportion with that of [Ca^2+^]_i_, which also suggests that cytosolic alkalinization or acidification are associated with changes of [Ca^2+^]_i_, but the specific interaction mechanism of pH_i_ and [Ca^2+^]_i_ in these conditions remains unclear, and need further researches to clarify.

## 3. Experimental

### 3.1. Reagents

The Rhodojaponin-III (98%) was isolated by using HPLC from the flowers of *R. molle* in the Laboratory of Insect Toxicology, South China Agricultural University. Fura-3/AM, Snarf1/AM were purchased from Sigma and stock solutions of all molecules were initially dissolved in dimethyl sulfoxide (DMSO), diluted to their final concentration in phosphate buffer solution (135 mM NaCl, 2.7 mM KCl, 1.5 mM KH_2_PO_4_, and 8 mM K_2_HPO_4_, pH 7.2) and stored at −20 °C until used. All other chemicals were from standard commercial sources and reagent grade or the highest purity.

### 3.2. Cell Culture

Sf9 cells were obtained from State Key Laboratory of Biocontrol, Sun Yat-sen University, and were cultured in Grace’s medium (GIBCO-BRL, Grand Island, NY, USA) supplemented with 10% fetal bovine serum (heat-inactivated) and 1% penicillin/streptomycin. Cells were cultured at 27 °C and subcultured every 3 days. Confluent cells with >95% viability (tested with Trypan blue exclusion) were used in all experiments.

### 3.3. Cell Growth Curve

Sf9 cells were seeded onto 24-well plates (2 × 10^4^ cells per well). When cells were adherent, at concentrations of 1, 10,100 and 200 μg/mL was added to treated cells for serial times (0, 12, 24, 48, 72 and 96 h). Cells that cultured with 0.1% DMSO at the same times were used as control. After harvested, survival cell number for each time point was counted using Trypan blue exclusion test with a standard haemocytometer. Growth curve was made after analyzing the data.

### 3.4. Flow Cytometry

Sf9 cells were seeded onto a 25 cm^2^ plastic tissue culture flask (2 × 10^5^ cells per flask). When the density was 1 × 10^6^ cells/mL, cells were treated with R-III at concentrations of 1, 10, 100 and 200 μg/mL for serial times (24, 48 and 72 h). After harvested, cells were re-suspended and washed twice with phosphate buffer solution (PBS) (137 mM NaCl, 2.7 mM KCl, 100 mM Na_2_HPO_4_, 2 mM KH_2_PO_4_, pH 7.2). After that, cells were fixed in cold 70% ethanol, and stored below −20 °C over night. Prior to analysis, ethanol was removed and fixed cells were washed twice with PBS (pH 7.2). Cells were re-suspended in PI solution (50 μg/mL PI, 0.1% Triton X-100, 0.1 mM EDTA, 50 μg/mL RNase A) and incubated for 30 min at room temperature. After that cells were processed in a FACSCalibur (Becton Dickinson, USA). At least 2.0 × 10^4^ cells were counted in each assay. The fraction of the total cell population presented in G2/M phase was obtained from DNA histograms using Cell Quest and Modfit Software (Becton Dickinson, USA). All cytometry experiments were performed on cells in log phase of growth. Cells that cultured with 0.1% DMSO at the same times were used as control.

### 3.5. Fluorescence Measurements

Cell treatment: [Ca^2+^]_i_ measurements from Sf9 cells were performed with the fluorescent Ca^2+^ indicator fluo-3/AM. Cells were cultured in 35-mm polystyrenetissue culture dishes (Nunc, Denmark) and washed twice with PBS, than loaded with 1 uM membrane-permeant acetoxymethyl ester of the dye (fluo-3/AM) for 45min at 37 °C. After dye loading, cells were washed twice with Dhanks solution (137.93 mM NaCl, 5.33 mM KCl, 4.17 mM NaHCO_3_, 0.441 mM KH_2_PO_4_, 0.338 mM Na_2_HPO_4_, 5.56 mM D-Glucose, pH 7.2). The pre-treatment measurement of pHi was carried out same as [Ca^2+^]_i_ measurements, except that the final concentration of dye solution were 10 μM (Snarf1/AM).

Fluorescence measurement: Fluo-3/AM and Snarf1/AM is one of the most suitable Ca^2+^and pH indicators for CLSM, and widely used to monitor [Ca^2+^]_i_ and pH_i_ in various cells. It can be excited by an argon ion laser at 488 nm, and its emitted fluorescence (at wavelengths 520 nm) increases with increasing [Ca^2+^]_i_ [41] or pH_i_. To measure the Fluo-3/AM and Snarf1/AM fluorescence, laser scanning confocal microscopy (Leica TCS SP2AOBS, Germany) was used to scan the cells with good silhouette and recorded fluorescence at intervals of 6 s for more than 400 s, and room temperature was kept in 20–23 °C during the experiments. According to the experimental design, drug was added from concentrated solutions with a pipette directly into the culture dishes through a small hole on top of the cuvette lid, and in control assays the same volume of DMSO was added. Results were analyzed using the Leica confocal software, and got time-dependent curves of calcium fluorescence signal. Although fluorescence recordings could not be calibrated to count the absolute value of [Ca^2+^]_i_ [22], [Ca^2+^]_i_ change could be shown by relative change of fluorescence intensity.

### 3.6. Statistical Analysis

Data analysis was carried out using SAS software (SAS Institute Inc.) and Microsoft Excel software. Differences between the treatments were determined by Tukey’s multiple range tests (P < 0.05 being considered significant).

## 4. Conclusions

R-III displayed strong inhibitory activity on the proliferation of Sf9 cells, and interfered with the Sf9 cell division cycle and arrested them in G2/M. In addition, R-III perturbed [Ca^2+^]_i_ homeostasis by inducing [Ca^2+^]_i_ influx or efflux in Sf9 cells in the presence of Ca^2+^-containing or Ca^2+^-free buffer solution. In these conditions, pH_i_ showed proportional changes with that of [Ca^2+^]_i_ on the whole. According to the results and discussion in this paper, we supposed that there was a certain linkage for change of intracellular calcium, cell cycle arrest, cell proliferation inhibition in Sf9 cells induced R-III and that cytosolic alkalinization or acidification shifts are associated with changes of [Ca^2+^]_i_ level in Sf9 cells induced R-III.
